# Diversity of Microbial Communities in Sediment from Yeosu Bay, Republic of Korea, as Determined by 16S rRNA Gene Amplicon Sequencing

**DOI:** 10.1128/mra.00363-22

**Published:** 2022-06-22

**Authors:** Jun-Ho Lee, Maheshkumar Prakash Patil, Jong-Oh Kim, Hee-Eun Woo, Kyunghoi Kim

**Affiliations:** a Research Vessel *Nara*, Pukyong National University, Busan, Republic of Korea; b Industry-University Cooperation Foundation, Pukyong National University, Busan, Republic of Korea; c Department of Microbiology, Pukyong National University, Busan, Republic of Korea; d School of Marine and Fisheries Life Science, Pukyong National University, Busan, Republic of Korea; e Department of Ocean Engineering, Pukyong National University, Busan, Republic of Korea; Montana State University

## Abstract

Monitoring natural variations in microbial diversity is crucial because microorganisms play a major role in the environmental processes in marine sediment. To evaluate the microbial diversity in Yeosu Bay sediment, 16S rRNA gene amplicon sequencing was performed. *Proteobacteria*, *Chloroflexi*, and *Bacteroidetes* were the predominant phyla in all sediment samples observed.

## ANNOUNCEMENT

The microbial communities in marine sediment make up a major portion of the biosphere and play a vital role in biogeochemical processes such as nutrient (carbon, nitrogen, and sulfur) and energy recycling, as well as mineralization (1). Particulate organic and inorganic matter accumulates on the sediment from seawater, and marine sediments cover two-thirds of the Earth’s surface. As a result, sediments seem to be the most common source of organic and inorganic substances. The presence of members of the phylum *Chloroflexi* in sulfide-rich sediment, an abundance of anaerobic bacteria in the oxygen-depleted zone of sediment, and nitrate-reducing microorganisms in organically enriched sediments all indicate a close relationship between the marine environment and microbiota (2, 3). According to research, microbial diversity and metabolisms are influenced by the local environment and seem to change rapidly in response to environmental shifts. As a result, studying microbial diversity is important in order to understand the richness and spatial variation of these microorganisms. In this study, we used 16S rRNA gene amplicon sequencing to explore the spatial microbial community richness of Yeosu Bay sediment.

Sediment samples were taken at a depth of 1.0 ft from the surface of the sediment in Yeosu Bay, Republic of Korea, in December 2021. Sediment samples were collected in a sterile high-density polyethylene (HDPE) bottle, stored in an icebox, and shipped to Macrogen, Inc. (Seoul, South Korea), for 16S rRNA gene amplicon sequencing (3). Total DNA was extracted as indicated by the manufacturer using the DNeasy PowerMax soil kit (Qiagen, USA). Using Herculase II Fusion DNA polymerase and the Nextera XT index kit v2 with the primers Bakt_805R and Bakt_341F, a 16S rRNA gene amplicon sequencing library was created following the manufacturer’s instructions. PCR for the targeted regions, V3 and V4, was carried out as stated before (4). The gene amplicons were sequenced using the Illumina MiSeq platform in 301-bp paired-end format at Macrogen. Next, before the denoising analysis, Cutadapt was used to remove the adapters and primers (5). To obtain the amplicon sequence variants (ASVs), the raw reads were processed using DADA2 (6). The standard processing steps in the DADA2 workflow were performed, including quality filtering, denoising, merging, ASV inference, and chimera removal. The taxonomic assignment was determined using BLAST against the NCBI 16S microbial database. Unless otherwise stated, default settings for software were applied throughout the analysis. Details of the samples and sequenced data are summarized in Table 1.

**TABLE 1:** Summary of sediment samples and sequencing results from Yeosu Bay

Parameter	Data for sample from:			
	Station 1	Station 2	Station 3	Station 4
	34°52.167′N, 127°48.088′E	34°43.212′N, 127°49.266′E	34°34.547′N, 127°54.019′E	34°25.191′N, 127°58.207′E
Water temp (°C)	17.8	17.7	17.7	16.8
Water depth (m)	15	15	18	40
No. of input reads	93,246	92,752	88,027	100,236
No. of filtered reads	85,813	84,829	79,599	90,519
No. of denoised forward reads	75,258	77,712	73,259	83,068
No. of denoised reverse reads	78,653	79,667	74,847	85,087
No. of merged reads	53,861	62,622	59,811	67,150
No. of nonchimeric reads	50,323	58,754	56,401	62,852
SRA accession no.	SRX14741299	SRX14741300	SRX14741301	SRX14741302

**FIG 1:**
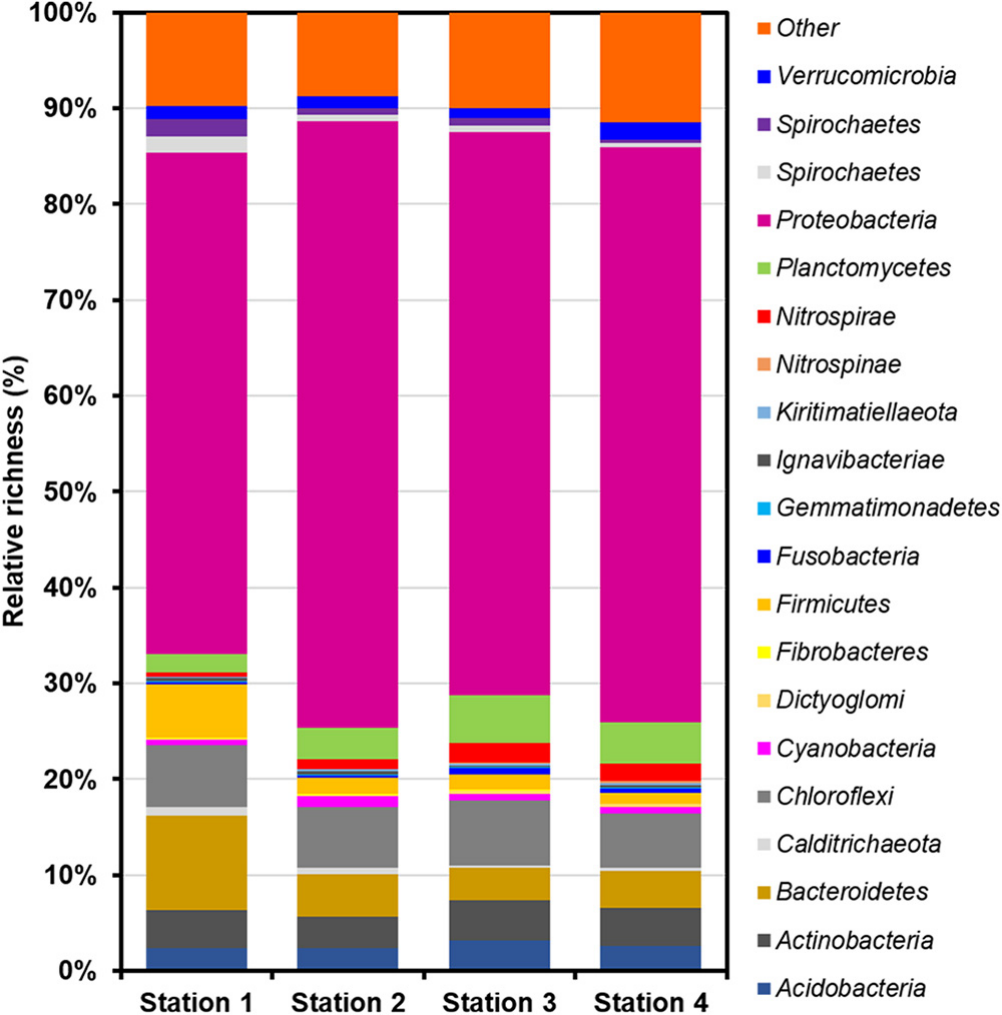
Relative richness of the sediment microbial communities in Yeosu Bay.

The phylum level richness of the microbial communities in the Yeosu Bay sediment is shown in Fig. 1; in all samples, *Proteobacteria* (53.10% to 63.61%) was predominant, followed by *Bacteroidetes* (3.36% to 10.04%) and *Chloroflexi* (5.66% to 6.90%). The present study findings could be an important resource for future research on the diversity of microbial communities in marine sediments.

### Data availability.

The sequence of the 16S rRNA gene amplicon acquired in this study has been submitted to the NCBI Sequence Read Archive (SRA) under the accession number PRJNA823394.
